# Effects of photobiomodulation (660 nm laser) on anthracycline extravasation: An experimental study

**DOI:** 10.1590/1518-8345.5786.3693

**Published:** 2022-10-17

**Authors:** Karina Alexandra Batista da Silva Freitas, Eliana Maria Minicucci, Valéria Flávia Batista da Silva, Benedito Donizete Menozzi, Hélio Langoni, Regina Célia Popim

**Affiliations:** 1 Universidade Estadual Paulista “Júlio de Mesquita Filho”, Faculdade de Medicina de Botucatu, Departamento de Enfermagem, Botucatu, SP, Brazil.; 2 Universidade Estadual de Mato Grosso do Sul, Unidade Universitária de Mundo Novo, Mundo Novo, MS, Brazil.; 3 Universidade Estadual Paulista “Júlio de Mesquita Filho”, Faculdade de Medicina Veterinária e Zootecnia de Botucatu, Botucatu, SP, Brazil.

**Keywords:** Low-Level Light Therapy, Extravasation of Diagnostic and Therapeutic Materials, Antineoplastic Agents, Wistar Rats, Nursing Care, Adverse Effects, Terapia Com Luz de Baixa Intensidade, Extravasamento de Materiais Terapêuticos e Diagnósticos, Antineoplásicos, Ratos Wistar, Cuidados de Enfermagem, Efeitos Adversos, Terapia de Luz de Baja Intensidad, Extravasación de Materiales Terapéuticos y Diagnósticos, Antineoplásicos, Ratas Wistar, Atención de Enfermería, Efectos Adversos

## Abstract

**Objective:**

to investigate the effect of using different agents (topical hyaluronidase, photobiomodulation, and the association of photobiomodulation with topical hyaluronidase) in preventing the formation of lesions caused by doxorubicin extravasation, as well as in the reduction of lesions formed by extravasation of this drug.

**Method:**

a quasi-experimental study conducted with 60 Wistar rats, randomized into four groups with 15 animals each. Group 1 (Control); Group 2 (Hyaluronidase); Group 3 (Photobiomodulation); and Group 4 (Hyaluronidase + Photobiomodulation). A wound was induced by applying 1 mg of doxorubicin to the subcutaneous tissue of the back of the animals. The concentration of topical hyaluronidase was 65 turbidity units/g and the energy employed was 1 joule of 100 mW red laser *per* square centimeter. With macroscopic evaluation every two days for 28 days, the following variables were observed: skin integrity, presence of blisters, hyperemia, exudate, bleeding, edema, crust, peeling and granulation tissue.

**Results:**

the animals from the groups subjected to photobiomodulation obtained better results in the assessment of the following variables: bleeding, hyperemia, exudate, intact skin and edema.

**Conclusion:**

it was evidenced that the association of photobiomodulation with topical hyaluronidase was effective in reducing the local effects and assisted in the wound healing process, and that PBM alone was able to prevent appearance of lesions.

## Resumo

**Objetivo:**

investigar o efeito do uso de diferentes agentes (hialuronidase tópica, fotobiomodulação e da associação da fotobiomodulação com a hialuronidase tópica) na prevenção de formação de lesões causadas por extravasamento de doxorrubicina bem como na diminuição de lesões formadas pelo extravasamento desta droga.

**Método:**

estudo experimental com 60 ratos Wistar, randomizados em quatro grupos de 15 animais. Grupo 1 (Controle); Grupo 2 (Hialuronidase); Grupo 3 (Fotobiomodulação) e Grupo 4 (Hialuronidase + Fotobiomodulação). Induziu-se ferida aplicando 1 mg de doxorrubicina no subcutâneo do dorso dos animais. A concentração da hialuronidase tópica foi de 65 unidades de turbidez/g, a energia empregada foi de 1 joule de laser vermelho 100 mW por centímetro quadrado. Com avaliação macroscópica a cada dois dias por 28 dias, observou-se as variáveis: integridade da pele, presença de flictema, hiperemia, exsudato, sangramento, edema, crosta, descamação e tecido de granulação.

**Resultados:**

os animais dos grupos com fotobiomodulação obtiveram melhores resultados na avaliação das variáveis: sangramento, hiperemia, exsudato, pele íntegra e edema.

**Conclusão:**

evidenciou-se que a associação da fotobiomodulação com a hialuronidase tópica foi eficaz na diminuição dos efeitos locais e auxiliou no processo de cicatrização da ferida e que a FBM isolada foi capaz de prevenir o aparecimento de lesões.

## Resumen

**Objetivo:**

investigar el efecto del uso de diferentes agentes (hialuronidasa tópica, fotobiomodulación y la combinación de fotobiomodulación y hialuronidasa tópica) en la prevención de la formación de lesiones causadas por la extravasación de doxorrubicina y en la reducción de las lesiones formadas por la extravasación de ese fármaco.

**Método:**

estudio experimental con 60 ratas Wistar, distribuidos aleatoriamente en cuatro grupos de 15 animales. Grupo 1 (Control); Grupo 2 (Hialuronidasa); Grupo 3 (Fotobiomodulación) y Grupo 4 (Hialuronidasa + Fotobiomodulación). La herida se indujo aplicando 1 mg de doxorrubicina por vía subcutánea en el lomo de los animales. La concentración de hialuronidasa tópica fue de 65 unidades de turbidez/g, la energía utilizada fue de 1 joule de láser rojo de 100 mW por centímetro cuadrado. En la evaluación macroscópica cada dos días durante 28 días se observaron las siguientes variables: piel intacta, presencia de flictena, hiperemia, exudado, sangrado, edema, costra, descamación y tejido de granulación.

**Resultados:**

los animales de los grupos con fotobiomodulación obtuvieron mejores resultados en la evaluación de las variables: sangrado, hiperemia, exudado, piel intacta y edema.

**Conclusión:**

se demostró que la combinación de fotobiomodulación y hialuronidasa tópica fue eficaz para reducir los efectos locales y ayudó en el proceso de cicatrización de heridas y que la FBM por sí sola previno la aparición de lesiones.

**Highlights**

(1) Using photobiomodulation proved to be effective in doxorubicin extravasation.

(2) Photobiomodulation can be considered a possible antidote for extravasation.

(3) It favors better quality of life in the patient, through a reduction in the formation of lesions.

(4) It exerts no deleterious effects on the body as some medications do.

(5) It is a low-cost therapy when compared to other antidotes.

## Introduction

Extravasation of antineoplastic agents (EA) is the most feared adverse event in Oncology, with the possibility of causing serious injuries, especially when the medication involved is classified as vesicant^1^. When infused outside a vessel, vesicant medications cause bubbles and necrosis. With a reported incidence in the literature from 0.1% to 6%, its main signs and symptoms are infusion stop, absence of venous return, burning sensation, hyperemia, edema and pain. As it is also considered as an emergency in Oncology, it requires a duly qualified and trained Nursing team for immediate treatment of this event. In view of this, it is necessary to investigate actions towards the prevention of complications referring to extravasation^1^ ^-^ ^2^. 

One of the most feared vesicant medications is doxorubicin (DOX), an anthracycline binding to Deoxyribonucleic Acid (DNA) that binds to the nucleic acids of tissues, generating free radicals, inhibiting the synthesis of local proteins and leading the tissue to chronic, progressive and increased necrosis, making the lesion deeper, more extensive and painful, exerting direct impacts on the patient’s quality of life. It can remain in tissues for up to 28 days, which requires constant evaluation^3^.

Currently, dexrazoxane is the antidote used in DOX extravasation. Its use is approved by the Food and Drugs Administration (FDA) and by the European Commission and has 98% efficacy. The mechanism of action is binding of the drug to the iron molecule preventing the formation of free radicals^4^. In addition to being unfeasible for the public sector (due to its price), it can cause side effects such as nausea, vomiting, mild pain at the infusion site and reversible increases in liver enzymes^2^ ^,^ ^5^.

Hyaluronidase is a widely used antidote in the extravasation of chemotherapeutics; however, it is specific for non-DNA-binding vesicants (vinca and taxane alkaloids), although it is a potential antidote for anthracyclines. This medication modifies tissue permeability by hyaluronic acid hydrolysis, promoting reabsorption of fluids and reducing the edema. This enzyme is an antidote recommended by the Oncology Nursing Society (ONS), which 1.0 ml subcutaneous application of 150 UI/ml to the extravasation area^1^ ^,^ ^6^ ^-^ ^8^. After ten minutes since application, an increase in diffusion of the overflowed liquid can be seen in an area from 3 to 5 times greater than in an untreated area. Tissue permeability is restored from 24 to 48 hours after applying the antidote^2^ ^,^ ^7^ ^,^ ^9^.

Photobiomodulation (PBM) consists in irradiating non-ionizing light and is classified according to its wavelength into visible red (622-780 nm) or infrared (780-1,500 nm). It is quite used to accelerate the healing process of various types of lesions, promoting improvements in the patients’ quality of life and speeding up the treatments^10^. When applying PBM, photochemical and photophysical reactions occur where excitation of the electrons that release adenosine triphosphate (ATP) used by the cells for development of their functions is stimulated^8^ ^,^ ^11^ ^-^ ^13^. 

For this, it is necessary that light is absorbed by chromophores, which are interrelated molecules (enzymes, cell membranes, extracellular substances) with the ability to absorb light. Cytochrome C Oxidase (CCO) is the main chromophore and is located in unit IV of the mitochondrial respiratory chain. CCO absorption mainly by the red wavelength leads to photodisconnection of nitric oxide (NO), resulting in a reduction of oxidative stress and, consequently, in an increase in the production of reactive oxygen species (ROS), ATP and Ca^2+^ ions, favoring the intended biological response (anti-inflammatory, analgesic, scarring) and increasing cell differentiation, proliferation and migration^11^ ^,^ ^13^.

The association between topical hyaluronidase 65 UTR/g and PBM is already used in an Oncology Outpatient service of a tertiary-level Public Hospital from the inland of the state of São Paulo. However, despite excellent results, the number of patients with extravasation is low, and there is no scientific evidence on this association. Consequently, it was necessary to conduct an experimental study to evidence this practice, thus justifying this study.

Therefore, the objective of this research was to investigate the effect of using different agents (topical hyaluronidase, PBM, and the association of PBM with topical hyaluronidase) in preventing the formation of lesions caused by antineoplastic DOX extravasation, as well as the reduction of lesions formed after extravasation of this drug.

## Method

### Type of study

An experimental study conducted with 60 albino Wistar adult female rats of the *Rattus norvegicus* species, aged between 3 and 4 months old. In randomized experimental studies, the researcher randomly assigns the subjects to a control group and to one or more experimental groups, minimizing selection bias, in addition to allowing isolating the effect of an intervention^14^.

### Study locus and period

The experiment was carried out from 08/15/2019 to 09/16/2019 at the Experimental Laboratory for Diagnosis of Zoonoses, Department of Veterinary Hygiene and Public Health, School of Veterinary Medicine and Zootechnics, UNESP, Botucatu-SP, Brazil.

### Population 

The animals were provided by the Creation Vivarium located in the Biotechnology Institute (*Instituto de Biotecnologia*, IBTEC) of the “Júlio de Mesquita Filho” State University of São Paulo. Immediately after that, they were kept in the Experimentation Vivarium for a 15-day adaptation period, and only then were they submitted to the experiment. Female rats were selected due to their differences regarding behavior and aggressiveness when compared to males, easing manipulation. Throughout the period, temperature, ammonia level and bed change were controlled, as well as water supply and *ad libitum* feed. They were clinically examined by a veterinarian at admission to the laboratory and during the entire experiment. In order to ensure environmental enrichment, paper rolls were places in the cages in an attempt to reduce the stress caused by manipulation and isolation.

### Selection and randomization criteria

To perform the randomization procedure, after the adaptation period, the animals were identified with numbers from 1 to 60, with a pilot pen in the dorsal region. Four envelopes were prepared and identified with the type of treatment, as follows:

*Envelope 1: Control Group (no antidote).*
*Envelope 2: Hyaluronidase Group (H).*
*Envelope 3: Photobiomodulation Group (PBM).*
*Envelope 4: Photobiomodulation + Hyialuronidase Group (PBM+H).*

Subsequently, one of the laboratory team members made a draw with numbers from 1 to 60. 

The first 15 animals drawn were allocated to Envelope 1, and so on.

Each group consisted of 15 animals that were housed in rectangular polypropylene cages with 5 animals each, placed on ALESCO^®^ ventilated racks, kept under room temperature and with free movement. It is important to note that, after DOX inoculation, the animals were separated, keeping them in individual cages. 

The cages were identified with the numbers corresponding to the animals and with the group to which they belonged.

### Sample definition

Considering that there is 50% occurrence of necrosis cases control) and assuming that application of the treatment reduces this percentage to zero (no necrosis), the minimum size for carrying out the experiment with a 5% margin of error, 95% compatibility and 90% power will be 15 animals per group (60 animals for all 4 groups).

### Study variables

The qualitative variables analyzed were related to the appearance of the extravasation site and to the formation or not of lesions such as: intact skin, blister (intact or ruptured), hyperemia (marginal, central, or generalized), exudate (absent, mild, moderate, severe), bleeding (absent, mild, moderate, severe), edema (absent, mild, moderate, severe), crust (absent and present: loosely adhered, very adhered), peeling (absent or present - mild, moderate, severe) and granulation tissue (present, absent).

### Instruments used for data collection

The process of wound formation was monitored by the researcher every 48 hours for 28 days, through an evaluation form that included presence of intact skin, blisters, hyperemia, exudate, bleeding, edema, crust, peeling and granulation tissue(15). At the same time, a database of photographic records (CANON Power Shot SX170IS digital camera with 1/250 speed and distance of 0.7) was prepared to assist in interpreting evolution of the wounds. Angle and distance of the photographs were calculated by a professional photographer from the Botucatu Medical School, in order to standardize the images. 

### Data collection

After the adaptation period, the animals were subjected to inhalational general anesthesia with isoflurane gas and trichotomy of the dorsal region with an electric trichotomizer and administered 1.0 mg of DOX (0.5 ml) in the subcutaneous tissue located between the scapula and the end of the ribs, using a graduated 1 ml syringe and a 13 G x 4.5 mm hypodermic needle. 

The DOX vial contained 10 mg in 5 ml, making up an equivalent of 2 mg/ml. 0.5 ml of the solution was aspirated, containing a total of 1 mg of DOX. A research study carried out with extravasation in rats proved that an intradermal injection of 1 mg of DOX was capable of causing necrosis^6^.

The hyaluronidase used in the study was manipulated at the *Cruz Vermelha* Pharmacy in Botucatu-SP, with a concentration of 65 UTR with 1 g base cream.

After DOX inoculation, the antidotes were applied as follows:

- Control Group (no antidote): no antidote was applied;
- Hyaluronidase Group (H): the topical hyaluronidase antidote was applied 15 minutes after inoculation and, daily, in the form of an ointment on the entire back of the animal, even in the presence of lesion;
- PBM Group: application of 100 mW PBM, 1 joule red light (10 seconds), wavelength of 660 nm, at the inoculation point occurred 10 minutes after administration of doxorubicin. In the subsequent days, in the animals that did not show lesions, 1 point of 1 joule (10 seconds) of PBM was performed at the inoculation point. When lesions appeared, in wounds up to 1 cm in area, a 1 joule point was irradiated (10 seconds), with a total energy of 1 joule in the center of the lesion; in wounds up to 2 cm in area, it was irradiated to the North, South, East and West (totaling 4 points - 40 seconds), with a total energy of 4 joules. Topical dressings with primary coverage were not performed, leaving the lesion area exposed.
- PBM+H Group: PBM was performed as reported in the PBM Group and, immediately after the laser, the hyaluronidase antidote was applied, as described in the H Group.

A pen-shaped semiconductor diode laser device was used (Therapy EC, DMC group, São Carlos, SP, Brazil), which emits two wavelengths: a laser diode at 660 nm (red) and a laser diode 808 nm (infrared). Peak emission power was 100 mW, in continuous mode delivery. The spacer at the end of the device was used, as well as a PVC film to avoid cross-contamination and wear of the appliance. 

The wound formation process was monitored by the researcher every 48 hours for 28 days and euthanasia was performed at day 30.

### Data treatment and analysis 

After data tabulation, a qualitative and descriptive analysis of the variables (bleeding, hyperemia, exudate, bleeding, edema, crust, peeling and granulation tissue) was performed by group (Control, Hyaluronidase, PBM and PBM+H) throughout the treatment days.

### Ethical aspects

The research was approved on 02/11/2019 by the Commission of Ethics in Use of Animals (*Comissão de Ética no Uso de Animais*, CEUA) at the Botucatu Veterinary and Zootechnics School - UNESP, with protocol No. 0026/2019.

## Results

It was observed that the groups that made use of hyaluronidase showed more animal losses (H: 09/15: 60% and PBM+H: 8/15: 53.3%). We believe that this loss can be related to several factors such as: toxicity generated by anthracycline, toxicity by hyaluronidase, and factors related to animal behavior. Although the groups were evaluated every two days, hyaluronidase was administered daily, with the possibility that such manipulation of the animals be considered stressing. 

**Figure 1:**
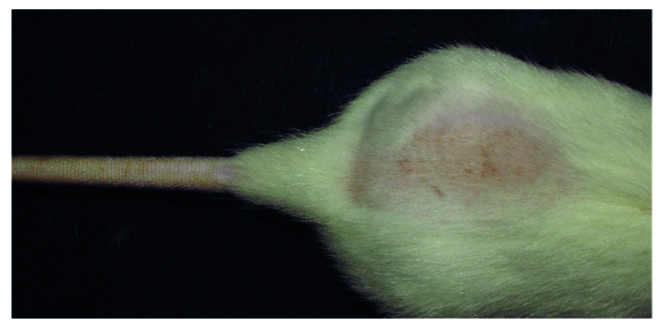
An animal from the PBM+H group at D28, without lesions in the back after doxorubicin extravasation. Botucatu, SP, Brazil, 2019

It was noticed that some animals from the PBM group (4/15: 26%) did not show lesions (Figure 1). In animals that presented lesions, it was observed that they started at D4, becoming more evident in group C (7/15: 46%) with the maximum time for wound formation at D8 for the photobiomodulation group. 

**Figure 2:**
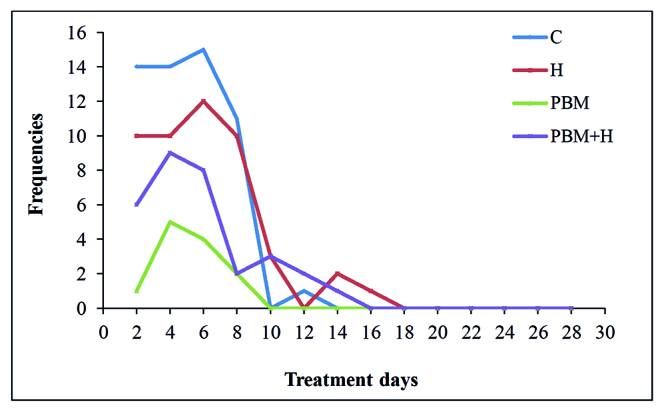
Frequency of animals with hyperemia after DOX inoculation in subcutaneous tissue, in all four groups and throughout the treatments. C = Control; H = Hyaluronidase; PBM = Photobiomodulation; PBM+H = Photobiomodulation + Hyaluronidase. Botucatu, SP, Brazil, 2019

Hyperemia was already observed at the first evaluation day (D2) in most of the animals analyzed from groups C and H, extending up to D16. It was observed that in the groups in which PBM was used, few animals presented hyperemia, with a maximum peak at D4 for both groups, with a lower frequency of animals involved, PBM (5/15: 33%) and PBM+H (9/15: 60%) (Figure 2).

Exudate was mostly found in Group C (14/15: 93%) starting at D10, reaching a maximum number of animals at D12 and extending until the end of the experiment. The animals that participated in the PBM and PBM+H groups did not show formation of exudate until the end of the experiment.

Of the animals from the groups with PBM, only one presented bleeding during the entire research. In turn, the animals from groups C (9/15: 60%) and H (7/15: 46.6%) showed bleeding at the third evaluation day (D6).

Crust was observed throughout the research in animals from all groups, although in a smaller number of animals from groups H, PBM and PBM+H.

**Figure 3:**
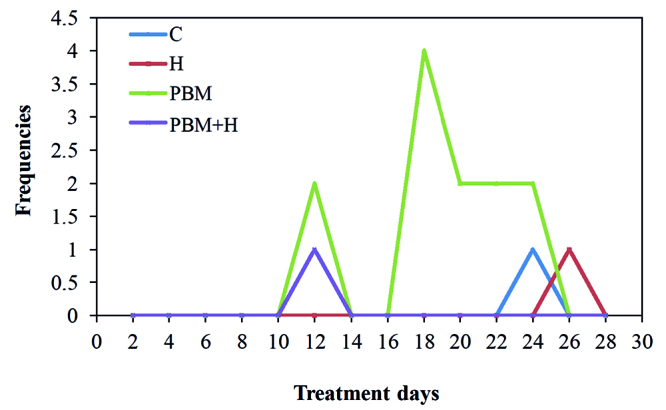
Frequency of animals with granulation tissue after DOX inoculation in subcutaneous tissue, in all four groups, throughout the treatments. C = Control; H = Hyaluronidase; PBM = Photobiomodulation; PBM+H = Photobiomodulation + Hyaluronidase. Botucatu, SP, Brazil, 2019

Figure 3 shows that granulation tissue was more found in animals from the PMB and PBM+H groups, as early as D12. The lesions were in the healing process, with replacement of granulation tissue by epithelium from D20 onwards.

The animals from Group C presented a higher rate of necrosis on all treatment days, starting on the fourth evaluation day (D4) and extending steadily and progressively throughout the study. The animals from the H, PBM and PBM+H groups also showed necrosis, although more superficial, in lower numbers, and with an improvement in the lesion at the end of the treatment.

**Figure 4:**
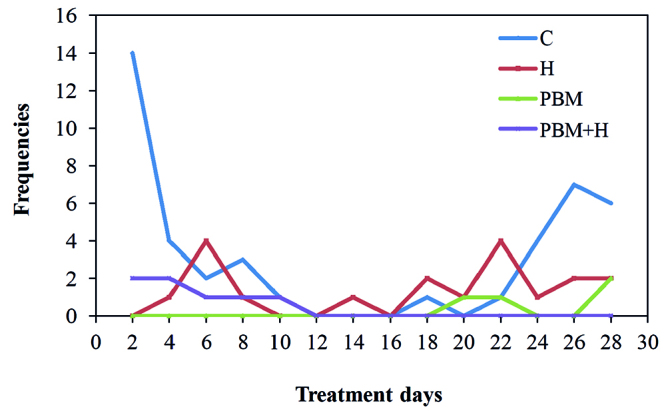
Frequency of animals with edema after DOX inoculation in subcutaneous tissue, in all four groups and throughout the treatments. C = Control; H = Hyaluronidase; PBM = Photobiomodulation; PBM+H = Photobiomodulation + Hyaluronidase. Botucatu, SP, Brazil, 2019

Edema was observed in 14 (93%) animals from the Control Group already at the first evaluation day (D2) (Figure 4). A lower number of animals from the PBM group showed edema (4/15: 26%) only at days 20, 22 and 28. On the other hand, the animals from the PBM+H group presented the best conditions in this aspect. Only two animals showed edema on the first two evaluation days, with only one of them maintaining the edema up to D10.

**Figure 5:**
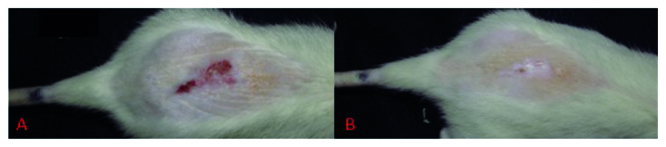
An animal from the PBM group at two moments: A: Presence of wound at D16; and B: Full healing of the lesion at D28. Botucatu, SP, Brazil, 2019

The healing process with epithelial tissue was observed at D12 with animals from the PBM+H group. It was observed that, after 28 days of evaluation, two animals from the PBM and PBM+H groups showed complete healing of the lesions. At the end of the study, six animals (40%) from the PBM group and two (13%) from the PBM+H presented intact skin (Figure 5).

## Discussion

There was premature death of 23 animals, which can be related to several factors, such as the toxicity generated by anthracycline and hyaluronidase and animal behavior. It was observed that the animals that died the most were from the groups that contained hyaluronidase. We infer that even when the ointment is being applied correctly to the back of the animal, due to its significant fluidity, it generated ease of licking and possible intoxication. 

Hyaluronidase has been widely used in ophthalmic and dermatological surgeries, as the enzyme degrades the hyaluronic acid matrix, increasing tissue permeability and favoring drug absorption. In addition to that, it is already widely used in EA management, subcutaneously^16^. Although we used a topical presentation in our research, no references to this application route in extravasation were found in the literature, nor on its toxic effects when ingested. 

The toxicity generated by anthracycline corresponds to the symptoms observed in the animals, such as diarrhea and weight loss. As it is non-selective, DOX causes various side effects, such as nausea, vomiting, diarrhea and anorexia with consequent weight loss^17^. In addition to these effects, the acute inflammation and oxidative stress caused by DOX can be related to toxicity in multiple organs^18^. We noticed that one animal from the Control Group showed ascites at the end of the study. The autopsy was performed after euthanasia, noticing significant adherence to the liver, spleen, heart and intestines.

Better preservation of skin morphology and reduction of edema and ulcer size were observed in an experimental study with Wistar rats that were administered an intradermal injection of vincristine and were treated with hyaluronidase^6^. This fact corroborates the results found in our study, in which only 2 animals from the PBM+H group presented edema. 

PBM also plays an important role in the prevention or reduction of edema during tissue repair processes since, after all, the benefits related to reduction of the edema, oxidative stress and pro-inflammatory cytokines are already well established^19^. This fact is related to a considerable increase in blood flow due to the vasodilating action of nitric oxide (NO); however, at the same time, lymphatic drainage is stimulated, reducing the edema through the control of pro-inflammatory cytokines^20^.

During this research, our study group published a retrospective cross-sectional study of the Oncology Outpatient service of a Public Hospital from the inland of the State of São Paulo, Brazil, with the objective of analyzing the effect of PBM and topical hyaluronidase on extravasation and infiltration of antineoplastic drugs in a 21-month period. 8 extravasations (among them 01 of anthracycline) and 7 infiltrations were reported. All were followed-up and received a mean of 2 PBM sessions (1 joule - 100 mw) and topical hyaluronidase (65 UTR) 3 times a day for 4 days. It was found that there was no formation of lesions, even in the patient who used anthracycline^21^. 

During DOX extravasation, there is cell death in the tissues underlying the extravasation site, with the release of free radicals triggering a marked oxidative stress, increasing the inflammatory process and causing necrosis^22^.

From photochemical reactions, PBM restores the cellular function through the release of reactive oxygen species, causing cellular balance restoration by the production of antioxidant enzymes and most importantly, without deleterious effects to the organism^4^. Unlike dexrazoxane, which, despite having a proven efficiency of 98%, also causes some side effects such as nausea, vomiting, mild pain at the infusion site and reversible increases in liver enzymes^1^ ^,^ ^5^ ^,^ ^23^. Unfortunately, it is unfeasible for many services due to its high cost. 

This is the first paper to investigate the association of PBM with topical hyaluronidase in DOX extravasation. However, the efficacy of PBM has already been proven in other types of lesion, even with the possibility of being associated with topical therapies. 

PBM is already quite used to heal wounds of various etiologies. In a study conducted to evaluate the effect of laser therapy at different wavelengths on the expression of growth factors and inflammatory mediators in the healing processes of pressure ulcers, it was observed that the wavelength of 658 nm was more effective. It is believed that this effect is associated with inhibition of the inflammatory processes^24^. 

In a case study on anthracycline (epirubicin) extravasation in a central venous access (fully implanted catheter) in the right hemithorax, it was observed that the patient showed necrosis in 2/3 of her breast in the first 10 days, lasting for 32 days when she underwent a surgical procedure to dry the necrosis. It is important to report that no antidote was applied to this patient, as 24 hours had already elapsed since extravasation. A skin graft was placed in the site and, after 8 months, the breast reconstruction procedure was performed^25^. A similar result was found in our study regarding the antidote and necrosis formation. In animals that did not receive the antidote, a greater amount of necrosis was observed at all moments evaluated, starting on the fourth evaluation day and extending steadily and progressively throughout the research; differently from what was found with the animals of the groups that used antidotes. All groups showed necrosis, although to a lesser extent and with improvement of the lesion and disappearance of necrosis at the end of the study.

In the study, we found that the granulation tissue was more evident in the animals from the PBM group, with onset of its appearance at D12, maximum peak at D18, stabilization from D20 and a sharp drop at D26, showing replacement of the granulation tissue by gradual epithelization with consequent wound healing of 2 animals from the PBM group and 2 animals from the PBM+H group. 

This study provides diverse evidence for using PBM associated with hyaluronidase in DOX extravasation, providing important and relevant information for the formulation of protocols by Oncology professionals. It has sufficient clinical relevance for the possibility of incorporating new technologies by the Unified Health System (*Sistema Único de Saúde*, SUS). 

### Study limitations

We consider the loss of some animals from the groups in use of hyaluronidase as a limitation of this study, which may have been due to toxicity. 

Another limiting factor is the non-existence of literature on photobiomodulation in anthracycline extravasation. We suggest that this technique be encouraged in several Health Institutions and that case reports be published in order to increase the evidence, as it would not be feasible to carry out experimental or clinical studies with extravasations in humans. 

### Advances for the Nursing/Health areas

This research can be considered the initial milestone in the use of photobiomodulation in anthracycline extravasation, ensuring professional autonomy for nurses in the prevention and treatment of such an important adverse event. 

New technologies should be incorporated in order to ensure better care for cancer patients.

## Conclusion

The results evidenced that the association of PBM (660 nm - 1 J) with topical hyaluronidase (65 UTR) on DOX extravasation was effective in reducing the local effects and assisted in the wound healing process, as well as that PBM alone was able to prevent the appearance of lesions. This therapy is a good alternative to treat extravasation, with the possibility of being incorporated into the clinical practice. 
